# The Institutional Worker

**Published:** 1921-01-22

**Authors:** 


### THE INSTITUTIONAL WORKER.

*Being a Special Supplement to "The Hospital."*

### OUR BUREAU OF INFORMATION.

***Rules for Correspondents.***

1. Every letter must be accompanied by the coupon to be cut from the back cover (inside page) of The Hospital, current issue, and must contain the name and address of the correspondent with pseudonym for publication if desired. Replies by post cannot be given save under exceptional circumstances at the Editor's discretion.

2. Letters from Approved Homes in reply to special needs published in the Bureau should state terms and full particulars, and be sent prepaid under cover to the Editor of the Bureau with name written across coupon for identification.

3. Proprietors of Homes which have not yet been entered on the List of Approved Homes, but have spare accommodation likely to suit special needs, are invited to write for an application form for registration. The fee for registration, which includes two announcements of the Home in the Bureau and other privileges, is 10s.

4. All communications to be addressed to the Editor of The Hospital, 28 Southampton Street, Strand, London, W.C. 2, and marked "Bureau of Information."

### INSTITUTION FACTS AND FIGURES.

### Nursing School Libraries.

We had hoped that the subject of nurses' libraries held a place of sufficient importance among our readers to extract some illuminating and useful contribution from their pens to these columns, and we confess to some disappointment with the replies we received to the November question. Some of the great training-schools for nurses are equipped with excellent libraries, but it is to be feared that too little attention is paid to this important department of the training-school at many of the smaller hospitals. We noticed on a recent visit to one of the smaller London general hospitals that an attempt had been made to found a library for the nurses. There were already on the shelves of a very handsome bookcase some four or five hundred volumes, but we were disappointed to find that the intention was apparently to provide for lighter reading only, and that much of the literature was of a character scarcely calculated to satisfy a person of average intelligence.

Mental recreation is very properly one of the purposes of a hospital nurses' library, for no one can at all times advantageously indulge in mental gymnastics. The ideal library should, however, provide an adequate supply of standard books selected for the requirements of the teacher as well as the student, and it should be a source of information on subjects associated with the school curriculum, as well as on topics of broader educational interest. While a good library is essential for the nurse in training, it should also serve as a place where her seniors may find food for intellectual exercise.

One of the first considerations before organising a library is to select a room suitable for the purpose. This is a matter which may present a real difficulty, for probably very few hospitals have a spare room to devote to this purpose. All difficulties can be solved, however, if properly tackled, and as a good library is an important aid in training, the matter is one which merits the attention of hospital managers. The library should be located in a quiet part of the building, and the room should be large enough to allow for expansion. Where the hospital is fortunate enough to possess a room set apart for lectures, this room might very well serve the dual purpose.

In furnishing, appearance and physical comfort are features not to be lost sight of, for a pleasing atmosphere is of some importance. For the accommodation of the books it is probably cheapest to fit shelves to the walls. The standard library shelf is three feet long and eight inches deep, and one inch is the usual thickness of the material used; the space between each shelf is ten inches. If provision be not made for adjustment, a few sections should be provided capable of holding larger books. A section of seven shelves will hold about 200 books. The sectional type of bookcase is no doubt the best, but furniture of this kind is now very expensive. For the rest, an oblong table, light comfortable chairs, and a librarian's desk placed in such a position as to permit of supervision of the room, complete the equipment.

The next matter for consideration is the provision of books and the means of upkeep, for a library must necessarily be a growing institution. Some definite system of expansion must be arranged and magazines and periodicals must be provided. For this a capital sum and a small annual income are obviously necessary. Need a scheme for the establishment of so useful a department be abandoned for lack of funds? We think not. A useful enterprise will always find its supporters, and we know more than one matron who can readily obtain funds for objects associated with the interests of the nursing profession. She can get help through the Ladies' Association and from the many friends whom her professional duties have gained for her. A newspaper appeal for a special purpose of this kind is often successful. A small annual subscription from each one making use of the library would provide an initial income and help considerably to stimulate outside support.

The management of the library should be in the hands of a small committee composed of persons who have special knowledge of the requirements.

The classification and cataloguing of books is a subject we will not attempt to elaborate. It must be borne in mind that the usefulness of the library depends largely upon the accessibility of the literature stored in it. The system adopted must depend upon the size of the library, and for one of the kind under discussion classification of subject and a cross-index catalogue (author, title, and subject) would probably be most suitable. This is a matter upon which we have no doubt the librarian of the local public library would gladly advise, as well as upon the method of recording books loaned, which is also an important feature.

The efficient management of a library requires the constant attendance of a librarian, for a good librarian can do much to help the student. Such an ideal arrangement is impracticable in the case of a small library. Nevertheless some arrangement should be made, possibly by the establishment of a roster of librarians, whereby the nurses may have free access to the books, and the resources of the library may be kept intact and in order.

**The Editor will be glad to receive correspondence and to consider contributions upon all subjects relating to institutional work which affect the welfare of institutional workers.**

### Question for January.

### CHECKS ON WASTE.

Describe methods for keeping a check on the use of drugs and dressings in the hospital wards and operating-theatre with a view to prevent waste.

**(A minimum payment of 10s. 6d. will be made for each published answer.)**

RULES.

The following rules must be observed:—

1. Contributions must be written on one side of the paper. Conciseness and terseness are desirable features. The MS. must bear the name and address of the sender and be accompanied by coupon to be cut from the back cover (inside page) of the current issue of THE HOSPITAL. A pseudonym must be chosen if the name is not to be published.

2. Contributions must be addressed to the Editor of THE HOSPITAL Institutional Worker Supplement, 28 & 29 Southampton Street, Strand, London, W.C. 2, should reach him before the end of the current month, and be marked in the left-hand corner "Facts and Figures."

### QUESTIONS INVITED.

IN connection with our Question Box, we offer every month five shillings for the best question sent in for consideration. The questions may be on any institutional subject and concern any department, from the secretary's to the porter's, or the matron's to the domestic staff; the one essential is that they must be practical and deal with points that have a definite relation to hospital or institutional administration, upkeep, finance, or management. Points relating to artificers' work, housekeeping, the laundry, out-patients, and other practical matters will be welcome. It is hoped that thus, when difficult or doubtful points arise in the course of their work, institutional workers will be encouraged to put them in the form of a question and send them to the Editor, THE HOSPITAL Bureau of Information, 28 Southampton Street, Strand, W.C. 2, marked "Question Box."

The best questions will be published in due course and our readers will be given the opportunity of answering them. Thus all institutional workers may be able to co-operate with the view of helping the work and smoothing the difficulties of each other. In short, we want the Question Box of the *Institutional Worker* to be freely used by every worker habitually.

### ENQUIRIES AND ANSWERS.

*EMPLOYMENT AND TRAINING.*

### Dispensing Course.

WRITE to the London College of Pharmacy, 36 Clapham Road, London, S.W., and ask for terms and full particulars of their course in dispensing. —K. L. W.

### Fever Training.

IF you are a fully-trained general nurse you can qualify for your fever examination by spending twelve months in a fever hospital recognised by the Fever Nurses' Association. If you write to the Secretary of the Fever Nurses' Association at 8 Western Road, Romford, Essex, he will give you full particulars of training, etc.—PEGGY.

*MISCELLANEOUS.*

### Concealment of Pipes.

THE exposure of all pipes is, as a rule, favoured for the reason that a pipe should be regarded as a modified part of a machine which may require attention at any time. The cracking of a pipe, whether for the supply of water or the removal of waste, may cause considerable trouble and involve unnecessary expense if the pipe be concealed in plaster or cement. While a network of piping will undoubtedly harbour dust, yet all pipes can be so placed that they can be readily cleaned. Some discretion in this matter is possible. All pipes are not alike, nor equally liable to damage or stoppage. Thus it would be necessary only to keep such parts on the surface as experience shows are likely to require periodical attention.—INQUIRER.

### The Value of the Rotary Machine.

THE present day cost of printing indicates the need for the more general use of the duplicating machine. The equipment of an office is incomplete without a duplicating machine of some kind, and in the office of a hospital or charitable institution, where the issue of circular letters is a part of the daily routine, it is indispensable. The disadvantages of the average hand duplicating machine are that the process is somewhat dirty and the stencils are apt to crack, with the result that few impressions are really perfect. It is, perhaps, for this reason that a great deal of work which could readily be done in the office is sent to the printers. A good rotary machine obviates these disadvantages. The machine provided by D. Gestetner, of 44 and 45 Farringdon Street, E.C. 4, is an exceptionally good machine. It is self-feeding, readily handled, and will produce from fifty to seventy perfect copies per minute without the wasting of a single sheet of paper. The stencils can be cleaned and stored for use on other occasions. An especially useful feature is that stencils of note-heading or special forms can be obtained from the makers. These stencils are prepared by photographic process, and with care will last almost indefinitely. Though the cost of the machine is somewhat high, it would nevertheless very soon pay for itself.—SECRETARY.

### Nurses' Clubs in London.

WE give you the addresses of the clubs you ask for: The Imperial Nurses' Club, 137 Ebury Street, S.W. 1; Norfolk Square Nurses' Club, 51 Norfolk Square, W. 2; and the Nurses' Lodge, Colosseum Terrace, Regent's Park, N.W.—NURSE.

### A Useful Fish Cleaner.

A NOVEL little device of American origin has recently been placed on the market, which should prove especially serviceable in the hospital kitchen. It is a machine for rapidly cleaning fish. In three parts, it consists of a scaling plate, a hook, and a scraper. On the scaling plate is a pair of clamps, which hold the fish securely. The hook is for insertion in the head to hold the fish during the process. The scraper, the most important part of the device, enables the operator to remove every scale with little effort and without cutting or tearing the skin of the fish. Concealed in the handle is a knife, which is used for the removal of the head and all fins, etc.—K. L. B.

### The Annual Inventory.

WE are of opinion that it is just as important to take stock of the whole of the contents of the hospital each year as it is in commercial houses. Every department and every ward should have its inventory, and these should be carefully checked at least once a year. Slackness in this department of work not infrequently involves unnecessary purchases through accidental shelving of articles in current use. It ought to be possible to account for the disposal of everything purchased, and in the case of instruments, furniture, utensils, etc., new articles should not be issued until the need for renewal or replacement has been satisfactorily explained and recorded.—A. T.

### Hostels for Professional Women.

THE Ladies' National Clubs, Ltd., whose office is at 104A Knightsbridge, have three branches in South Kensington, one in Kensington, and one in Bayswater. There are also branches at Bexhill, St. Leonards, Folkestone, Eastbourne, Tunbridge Wells, and Walton-on-Thames. These clubs provide furnished and unfurnished "flatlets" for gentlewomen and married couples, who must be members. The rents vary from £3 3s. to 12s. 6d. weekly, unfurnished, and include rates, electric light, baths, constant hot water, use of general dining-room and lounge, with periodicals and papers, boxroom space, and separate larder. Meals can be taken in the restaurant or in individual "flatlets." The qualification for a member is an entrance fee of one guinea and an annual subscription of the same amount.—D. M. D.

